# MicroRNA-Mediated Responses to Chromium Stress Provide Insight Into Tolerance Characteristics of *Miscanthus sinensis*

**DOI:** 10.3389/fpls.2021.666117

**Published:** 2021-06-23

**Authors:** Gang Nie, Zongchao Liao, Minyi Zhong, Jie Zhou, Jiabang Cai, Aiyu Liu, Xia Wang, Xinquan Zhang

**Affiliations:** Department of Forage Science, College of Grassland Science and Technology, Sichuan Agricultural University, Chengdu, China

**Keywords:** chromium, high-throughput sequencing, microRNA, abiotic stress, *Miscanthus sinensis*

## Abstract

Chromium (Cr) is a heavy metal in nature, which poses a potential risk to toxicity to both animals and plants when releasing into the environment. However, the regulation of microRNA (miRNA)-mediated response to heavy metal Cr has not been studied in *Miscanthus sinensis*. In this study, based on high-throughput miRNA sequencing, a total of 104 conserved miRNAs and 158 nonconserved miRNAs were identified. Among them, there were 45 differentially expressed miRNAs in roots and 13 differentially expressed miRNAs in leaves. The hierarchical clustering analysis showed that these miRNAs were preferentially expressed in a certain tissue. There were 833 differentially expressed target genes of 45 miRNAs in roots and 280 differentially expressed target genes of 13 miRNA in leaves. After expression trend analysis, five significantly enriched modules were obtained in roots, and three significantly enriched trend blocks in leaves. Based on the candidate gene annotation and gene ontology (GO) and Kyoto Encyclopedia of Genes and Genomes (KEGG) function analysis, miR167a, novel_miR15, and novel_miR22 and their targets were potentially involved in Cr transportation and chelation. Besides, miR156a, miR164, miR396d, and novel_miR155 were identified as participating in the physiological and biochemical metabolisms and the detoxification of Cr of plants. The results demonstrated the critical role of miRNA-mediated responses to Cr treatment in *M. sinensis*, which involves ion uptake, transport, accumulation, and tolerance characteristics.

## Introduction

Heavy metal pollution of soil could lead to losses in agricultural yields and potentially affects human health when it enters the food chain, which has become one of the major ecological problems worldwide (Islam et al., 2014). Chromium (Cr) is a heavy metal by nature with trivalent Cr (III) and hexavalent Cr (VI) as two major stable chemical forms, which poses a potential risk to toxicity to both animals and plants when releasing into the environment (Tiwari et al., 2013). Cr (VI) compounds are known to have stronger oxidizing activity than Cr (III), and excessive accumulation in plants can cause extremely deleterious effects on morphological, physiological, and biochemical processes of plants, such as inhibition of seed germination and plant growth, damage of cell ultrastructure, induction of oxidative stress, and uptake of mineral nutrition (Rout et al., 2000; Dube et al., 2003; Choudhury and Panda, 2005; Shanker et al., 2005; Panda, 2007; Diwan et al., 2010). Previous studies found that *Glycine max* had a significant translocation of Cr to the aboveground plant parts (Mei et al., 2002). Besides, *Spirodela polyrhiza*, *Hydrodictyon reticulatum*, and *Ceratophyllum demersum* could effectively reduce about 90% of the Cr concentration of water (Rai et al., 1995). Goupil et al. (2009) detected that heat shock proteins (HSPs), metallothioneins (MTs), and GR isoforms were upregulated in tomato (*Lycopersicon esculentum*) when subjected to Cr stress. In Cr-stressed *Typha angustifolia*, a significant induction in the expression of ATP synthase, Rubisco small subunit, and coproporphyrinogen III oxidase (Bah et al., 2010) was detected. In short, to manage Cr contamination and limit Cr accumulation in crops, it is necessary to gain a better understanding of the uptake, transportation, and sequestration of Cr and of the adaptive response of plants to the stress.

*Miscanthus sinensis* is a vigorous C4 perennial grass that originates from East Asia, which is widely cultivated as forage, ornamental grass, and energy crop worldwide (Clifton-Brown and Lewandowski, 2002; Heaton et al., 2004). *Miscanthus sinensis* could develop a dense net of fibrous roots that contribute to broad adaptation, high water-use efficiency, and great resistance to drought, salt, and heavy metal stress (Ye et al., 2005; Ezaki et al., 2008; Nie et al., 2017). Besides, it has been reported that *M. sinensis* could effectively absorb and accumulate the Cr (Zhu et al., 2010; He et al., 2013; Liang et al., 2014), and the contaminated biomass could be burnt for bioenergy production with great commercial values (Arduini et al., 2006). On the other hand, about 99% of the Cr taken up by the *Miscanthus* plant was retained by the hypogeal part (Arduini et al., 2006). Thirty-six proteins were identified to be differently expressed in *M. sinensis* in response to Cr stress, including oxidative stress-related proteins and metabolism-related proteins (Sharmin et al., 2012). However, the molecular regulation network of Cr detoxification and tolerance characteristics of *M. sinensis* remain unknown due to the dearth of omic information.

Generally, plant responses to metal toxicity are regulated at both transcriptional and posttranscriptional levels (Mendoza-Soto et al., 2012). MicroRNAs (miRNAs) are a class of noncoding small regulatory RNAs with the length of 20–22 nt, which bind to mRNA and participate in regulating gene expression *via* histone modification and DNA methylation (Diederichs and Sven, 2011; Khraiwesh et al., 2012; Tiwari et al., 2013), and they play a critical role in the whole growth period or fall into an environmental stress in recent studies (Noman and Aqeel, 2017). In rice (*Oryza sativa*), several miRNAs, such as miR-156, miR-169, miR-171, miR-199, miR-396, miR-398, miR-399, and miR-408, had been reported as responsive miRNAs under cadmium and arsenic (Tang et al., 2014; Sharma et al., 2015). A high-throughput small RNA sequencing approach revealed that miR159, miR160, miR319, miR396, and miR390 were downregulated in response to Al exposure in *Medicago truncatula* (Chen et al., 2012). In addition, *M. truncatula* Hg-responsive miRNAs, such as miR167, miR172, miR169, miR164, and miR395 families, were identified (Zhou et al., 2011). However, few studies on Cr-responsive miRNAs have been reported in recent years. In rice, 13 conserved miRNAs were expressed preferentially in response to Cr stress, namely, miR156, miR159, miR160, miR166, miR169, miR171, miR396, miR397, miR408, miR444, miR1883, miR2877, and miR5072 (Dubey et al., 2020). Fifty-four differentially expressed known miRNAs and 16 novel miRNAs (*Raphanus sativus* L.), such as miR156, miR164, miR167, miR171, miR396, and miR399, were identified in radish (Liu et al., 2015). In tobacco (*Nicotiana tabacum*), 53 known miRNAs and 29 unknown miRNAs, including miR156, miR159, miR166, miR167, miR171, miR396, and miR399, were identified (Bukhari et al., 2015).

Given the fact that miRNAs are key regulators, they serve as the core of gene regulatory networks response to heavy metal stress. Therefore, in this study, Illumina HiSeqTM2500 platform was employed for the miRNA sequencing on *M. sinensis* plant subjected to Cr treatment. The aim of this study is to identify Cr-responsive miRNA and primary potential targets and explore the possible pathway that is involved in tolerance to Cr stress. The results established in this study would help to further elucidate the molecular mechanisms of *M. sinensis* in response to Cr stress and provide valuable bioenergy resources for the development of soil phytoremediation strategies.

## Materials and Methods

### Plant Materials

The *M. sinensis* plant material (M20100819) was provided by Sichuan Agricultural University and was collected from Ya’an, Sichuan (N 29°57'30.1'', E 102°24'36.4', 1,388 m). The seedlings were planted in a round plastic pot (14.3 cm × 11 cm × 11 cm) with a matrix of quartz sand and were irrigated by a nutrient solution of 1× Hoagland under a constant temperature (28°C for light/25°C for darkness with cycles of 12/12 h) and humidity (75%) in a growth chamber. According to the preliminary experiment, the morphology of *M. sinensis* was completely established after 4 months (Nie et al., 2017), and the phenotypic change was most obvious under 200 mg/L of Cr stress based on the study by Arduini et al. (2006). Therefore, after 4 months of growth, 15 pots were treated with 200 mg/L Cr (VI; K_2_Cr_2_O_7_) solution. After Cr treatment, the samples of roots and leaves (signed for MR and ML) were collected at 0, 12, 24, and 72 h for miRNA sequencing (MR0 and ML0, MR12 and ML12, MR24 and ML24, and MR72 and ML72, respectively), and all of these samples were quickly deep-frozen in a container with liquid nitrogen and then stored at −80°C for later RNA extraction. Each sample has three replicates.

### RNA Extraction and High-Throughput Sequencing

Total RNA was extracted from samples using the Direct-zol™ RNA MiniPrep Kit (Zymo Research Co., CA, United States), following the instructions of the manufacturer. Twenty-four RNA samples (MR0 and ML0: roots and leaves at 0 h, nontreated; MR12 and ML12: roots and leaves with Cr treated for 12 h; MR24 and ML24: roots and leaves with Cr treated for 24 h; and MR72 and ML72: roots and leaves with Cr treated for 72 h) were sent to the Tianjin Novogene Bioinformatic Technology Co., Ltd., (TianJing, China) for sequencing using the Illumina HiSeqTM2500.

### Establishment of Small RNA Library and Bioinformatic Identification of miRNAs

An equal amount of RNA (RIN number > 7.0) per sample was used to construct small RNA libraries using NEBNext® Multiplex Small RNA Library Prep Set for Illumina® (NEB, United States), following the recommendations of the manufacturer. The 3' and 5' end adapters were ligated to the two ends of miRNAs. Then, the first strand cDNA was synthesized using M-MuLV Reverse Transcriptase (RNase H–). Long Amp Taq 2X Master Mix, SR Primer for Illumina, and index (X) primer were used for PCR amplification. The PCR products were further purified on 8% polyacrylamide gel (100 V, 80 min). The DNA fragments corresponding to 140–160 bp were recovered and dissolved in 8-μl elution buffer. Finally, the library quality was assessed on the Agilent Bioanalyzer 2100 system using DNA high-sensitivity chips. Small RNA sequencing was performed on the Illumina HiSeqTM2500 platform to generate raw reads. The clean reads were obtained by removing reads containing poly N, with 5' adapter contaminants, without 3' adapter or the insert tag, containing poly A or T or G or C, and containing low quality and those smaller than 18 nt reads from the raw data. The small RNA tags were mapped to the *Sorghum bicolor* reference sequence (ftp://ftp.ensemblgenomes.org/pub/plants/release-41/gff3/sorghum *bicolor*; Langmead et al., 2009) without mismatch to analyze their expression and distribution on the reference. The mapped small RNA tags were used to identify known miRNA by aligning against the miRbase 20.0. The custom scripts were used to obtain the miRNA counts and base bias on the first position of an identified miRNA with certain length and on each position of all identified miRNAs, respectively. The characteristics of the hairpin structure of miRNA precursor can be used to predict novel miRNAs. The miREvo (Wen et al., 2012) and mirdeep2 (Friedlnder et al., 2012) software were integrated to predict novel miRNAs. The known miRNA used miFam.dat^1^ to identify families, and novel miRNA precursors were further submitted to Rfam^2^ to identify Rfam families.

### Differential Expression Analysis of miRNAs

According to the methods studied by Zhou et al. (2010), the miRNA expression levels were estimated by transcript per million (TPM). TPM avoided the effect of quantitative accuracy and normalized the expression level of small RNAs in different sequencing amounts. TPM was calculated as follows:

$$\mathrm{TPM}=\left(\mathrm{readCount}\times 1,000,000\right)/\mathrm{libsize}$$

(libsize: Sum of miRNA ReadCount of sample)

The data from the TPM normalization were used to identify the differentially expressed miRNAs based on the number of genomic tags in each sample and compare the miRNA abundance among the three sets of libraries. The fold change was calculated as follows:

$$\mathrm{Fold change}=\mathrm{log2}\;\left(\mathrm{miRNA}\;\mathrm{TPM}\;\mathrm{in}\;A/\mathrm{miRNA}\;\mathrm{TPM}\;\mathrm{in}\;B\right)$$

A: a sample of A time.

B: a sample of B time.

The positive and negative values indicated upregulation and downregulation of small RNA, respectively. The Benjamini–Hochberg method was used to adjust the *p* value. Value of *p* < 0.05 was set as the threshold for significantly differential expression. To identify differentially expressed miRNAs among Cr treatment at different points in time, heat maps were generated using Novomagic tools.

### Target Gene Prediction, Gene Ontology, and Kyoto Encyclopedia of Genes and Genomes Function Analysis

The target gene prediction of miRNAs was performed by psRobot_tar in psRobot (Wu et al., 2012). NovoMagic program^3^ was used to draw the Venn diagram. After finding the target genes of miRNAs, the same program was used for the GO and Kyoto Encyclopedia of Genes and Genomes (KEGG) enrichment analysis. The target genes were divided into three parts using the GO function package of R-Studio Team as follows: biological processes, molecular functions, and cellular components. The trend analysis was performed using the OmicShare tools.^4^ The small RNA sequencing data set was deposited into the NCBI database under accession number PRJNA595773.

### Verification of miRNA by qRT-PCR Analysis

Six Cr-responsive miRNAs, i.e., miR156a, miR167a, miR396d, miR5564a, novel_miR149, and novel_miR32, were selected for quantitative real time PCR (qRT-PCR) analysis (Table 1). The miRNA tailing reverse transcription primer was used for the transcription of total RNA, which was designed according to the method of Shi and Chiang (Rui and Chiang, 2005). Reverse transcription was performed using the Mir-X miRNA First-Strand Synthesis Kit (Clontech Laboratories, Inc., Shanghai, China) according to the instructions of the manufacturer. The cDNA (i.e., samples were same with sequence) was then used for qRT-PCR using specific forward primers (Table 1) and mRQ 3' universal reverse primers (10 μM). The 18S rRNA was used as an internal reference for tailing qRT-PCR. The qRT-PCR was performed using a Bio-Rad CFX96 Touch (Bio-Rad, Hercules, CA, United States). Each 20 μl reaction contained 10 μl 2× miRNA qPCR master mix, 0.8 μl (10 μM) forward primer, 0.8 μl (10 μM) reverse primer, 6.4 μl RNase-free water, and 2 μl cDNA template. The PCR procedure was carried out at 95°C for 30 s, followed by 40 cycles at 95°C for 5 s and at 60°C for 20 s. After the reaction was completed, a melting curve was set to evaluate primer specificity. The relative expression levels were calculated using the 2^−△△CT^ method (Livak and Schmittgen, 2001).

**Table 1:** Primers information of qRT-PCR verification for selected six *Miscanthus sinensis* miRNAs.

miRNA ID	Mature sequence	Forward primer
miR156a	UGACAGAAGAGAGUGAGCAC	GCCTGACAGAAGAGAGTGAGCAC
miR167a	UGAAGCUGCCAGCAUGAUCUA	GTGAAGCTGCCAGCATGATCT
miR396d	CUCCACAGGCUUUCUUGAACUG	CTCCACAGGCTTTCTTGAACTG
miR5564a	UGGGGAAGCAAUUCGUCGAACA	TGGGGAAGCAATTCGTCGAA
novel_miR149	AGUCCCGGCUGGUAUUACCAA	AGTCCCGGCTGGTATTACCAA
novel_miR32	CAGGCGUAGAGAAAACCGG	GCAGGCGTAGAGAAAACCGG

## Results

### Data Analysis of Small RNA Sequences

After sequencing with previously cultured *M. sinensis* materials that were grown under 0 h Cr treatment (ML0 and MR0), 12 h Cr treatment (ML12 and MR12), 24 h Cr treatment (ML24 and MR24), and 72 h Cr treatment (ML72 and MR72), a total of 24 small RNA libraries were established to identify and predict the miRNAs associated with Cr treatment. The sequences of shortages, low quality tags, *N* > 10% reads, adaptors, and adaptor–adaptor ligation were filtered out, and 355,453,701 clean reads were obtained (Supplementary Table S1). About 0.52–1.04 Gb raw reads were obtained from small RNA sequencing, and the averages of Q20 and Q30 were more than 99.55 and 98.74%, respectively. The miRNA length ranging from 18 to 30 nt was further mapped to the reference sequence using Bowtie software, with the average mapping rate of 62.20%.

### Identification of Cr-Responsive Differentially Expressed miRNAs in *M. sinensis*

**Figure 1:**
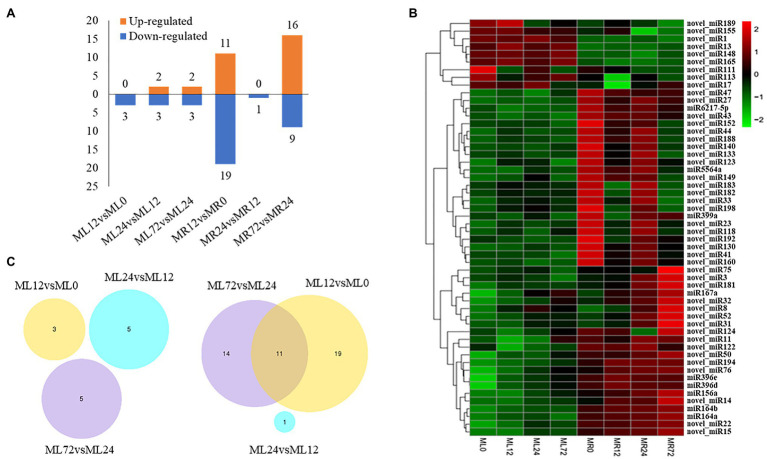
The identified differentially expressed microRNA (miRNA) response to Chromium (Cr) stress. **(A)** The histogram showed the number of upregulated and downregulated differentially expressed miRNAs caused by Cr treatment of six comparison groups. **(B)** Heat map of the expression levels of 54 differentially expressed miRNAs. Different colors indicate different expression levels, as shown in the scale. **(C)** Venn diagram showed the number of differentially expressed miRNAs in different comparison groups.

A total of 104 known miRNAs (Supplementary Table S2) were identified in 24 libraries, and 158 novel miRNAs (Supplementary Table S3) were predicted, which belong to 35 miRNA families. Among them, 54 differentially expressed miRNAs were detected (in comparison group: ML12 vs. ML0, ML24 vs. ML12, ML72 vs. ML24, MR12 vs. MR0, MR24 vs. MR12, and MR72 vs. MR24), including nine known miRNAs and 45 novel miRNAs, and four miRNAs (i.e., novel_miR149, novel_miR15, novel_miR22, and novel_miR32) are commonly identified in two plant organizations (Table 2). Out of 45 novel miRNAs, 13 were identified in the three comparison groups of leaves. In the root, there were 45 differentially expressed miRNAs, i.e., nine known miRNAs and 36 novel miRNAs. After Cr stress, 31 miRNAs were upregulated, 38 miRNAs were downregulated, and the most dramatic change occurred at the roots (Figure 1A). The hierarchical clustering analysis showed that these miRNAs were preferentially expressed in a certain tissue, most of which were highly expressed in the root tissue (Figure 1B). Especially, the expression trend of novel_miR15 and novel_miR22 is consistent in roots and leaves, and these two miRNAs could cluster together in a heat map. In addition, novel_miR15 and novel_miR22 clustered with the miR164 family. According to the expression of known miRNA, the miRNAs from the same family had similar expression pattern, for example, both miRNAs from miR396 family and miR164 family were upregulated in the root after the Cr stress. Furthermore, the Venn diagrams were used to compare and analyze the miRNAs differentially expressed at six comparison groups (Figure 1C). Among them, no common miRNA was found in these three groups of leaves, while there were 11 differentially expressed miRNAs (i.e., novel_miR14, novel_miR118, novel_miR32, novel_miR15, miR156a, miR167a, novel_miR182, novel_miR140, novel_miR183, novel_miR33, and novel_miR8) in the roots shared by MR12 vs. MR0 and MR72 vs. MR24. Interestingly, only one differentially expressed miRNA was identified in MR24 vs. MR12 comparable group (novel_miR155), indicating rare response at posttranscriptional levels to Cr stress during this stage.

**Table 2:** Numbers of differentially expressed miRNA targets in leaf and root organizations.

Number	Location	miRNA	target-number	Number	Location	miRNA	target-number
1	Common	novel_miR149	86	28	Root	novel_miR188	129
2	Common	novel_miR15	133	29	Root	novel_miR192	99
3	Common	novel_miR22	102	30	Root	novel_miR194	0
4	Common	novel_miR32	167	31	Root	novel_miR198	119
5	Leaf	novel_miR1	140	32	Root	novel_miR23	107
6	Leaf	novel_miR111	140	33	Root	novel_miR27	204
7	Leaf	novel_miR113	98	34	Root	novel_miR3	115
8	Leaf	novel_miR13	124	35	Root	novel_miR31	123
9	Leaf	novel_miR148	159	36	Root	novel_miR33	136
10	Leaf	novel_miR155	153	37	Root	novel_miR41	92
11	Leaf	novel_miR165	126	38	Root	novel_miR43	146
12	Leaf	novel_miR17	123	39	Root	novel_miR44	211
13	Leaf	novel_miR189	137	40	Root	novel_miR47	95
14	Root	novel_miR11	108	41	Root	novel_miR50	101
15	Root	novel_miR118	212	42	Root	novel_miR52	126
16	Root	novel_miR122	175	43	Root	novel_miR75	163
17	Root	novel_miR123	109	44	Root	novel_miR76	138
18	Root	novel_miR124	84	45	Root	novel_miR8	126
19	Root	novel_miR130	130	46	Root	miR156a	172
20	Root	novel_miR133	86	47	Root	miR164a	137
21	Root	novel_miR14	0	48	Root	miR164b	140
22	Root	novel_miR140	123	49	Root	miR167a	130
23	Root	novel_miR152	111	50	Root	miR396d	112
24	Root	novel_miR160	77	51	Root	miR396e	133
25	Root	novel_miR181	113	52	Root	miR399a	139
26	Root	novel_miR182	90	53	Root	miR5564a	149
27	Root	novel_miR183	83	54	Root	miR6217a-5p	121

### Prediction of Target Genes of the Differentially Expressed miRNAs

A total of 5,451 target genes of 54 differentially expressed miRNAs were predicted, and each miRNA was annotated to 123 genes on average (Table 2). The most abundant novel_miR118 corresponded to 222 target genes. However, novel_miR14 and novel_miR194 were not annotated to any target genes. Among them, 3,215 target genes were also differentially expressed in the transcriptome sequencing analysis after Cr treatment (|Fold change| > 1, FDR < 0.05, the data set was deposited into the NCBI database with accession number PRJNA594536, but not yet published).

**Figure 2:**
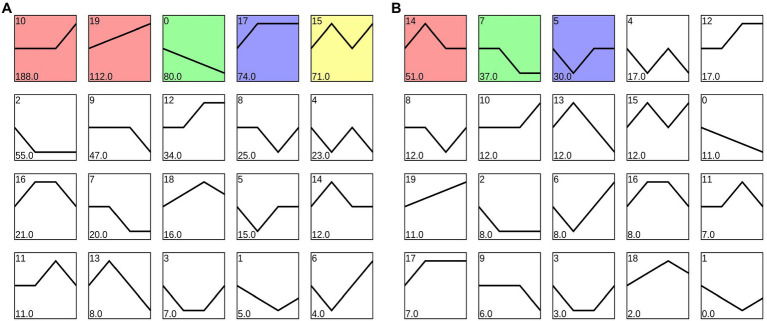
Trend analysis of the differentially expressed target genes of specific miRNAs in **(A)** roots and **(B)** leaves, and profiles are ordered based on the number of genes assigned.

A total of 833 differentially expressed target genes of 45 miRNAs were identified in roots. After trend analysis, five significantly enriched modules were obtained showing various expression trends; including continuous up- and downregulating in roots (Figure 2A; Supplementary Table S4). The number of genes in profile 10 was 188, which were the target genes of 37 miRNAs. Profile 10 enriched the most differentially expressed target genes in roots under Cr stress. Among them, there is a sulfate transmembrane transporter (Cluster-61280.0), which was targeted by miR167a. Besides, genes in profile 17 were 74, which were the target genes of 40 miRNAs, and a Mg^2+^ transporter protein (i.e., Cluster-42176.0, targeted by novel_miR22) and two ABC transporter proteins (i.e., Cluster-86748.1466 was the target gene of novel_miR15, and Cluster-25283.0 was the target gene of novel_miR3 and novel_miR8) were detected in this study. Interestingly, a *M. sinensis* MT-like protein gene (i.e., Cluster-86748.41948 in profile 6) was also the target gene of novel_miR15.

In addition, the genes in profile 19 were 112, which were the target genes of 41 miRNAs, and genes in profile 19 showed a continuously upregulated expression trend. An activated mitogen-activated protein kinase (Cluster-15974.0) was targeted by miR396d, a probable leucine-rich repeat receptor-like serine/threonine-protein kinase (Cluster-86748.44739) was targeted by miR396e, and a pyruvate dehydrogenase kinase (Cluster-86748.49811) was targeted by novel_miR44, which can participate in the signal transduction in plants after Cr stress through phosphorylation. In profile 0, the number of genes was 80, which were the target genes of 32 miRNAs. A cytochrome P450 gene (Cluster-86748.26078 was the target gene of miR156a) and an auxin-responsive protein (Cluster-86748.61059, target by novel_miR124) were detected, which potentially participated in the plant oxidation–reduction process and showed continuous downregulating expression trend in the block. There were 71 target genes in profile 15, which were the target genes of 32 miRNAs, and a glutaminyl-peptide cyclotransferase (i.e., Cluster-56602.0 was the target gene of miR156a) was detected in this study.

In leaves, the number of differentially expressed target genes of 13 miRNAs was 280, and three significantly enriched trend blocks were obtained after the trend analysis (Figure 2B; Supplementary Table S5). The number of genes in profile 14 was 51, including a MT protein gene (i.e., Cluster-86748.38482 was targeted by novel_miR155) participating in metal ion binding, one transcription factor of MYB (i.e., Cluster-86748.56774 was targeted by novel_miR17), and one transcription factor of bHLH129 isoform X2 (i.e., Cluster-86748.36885 was targeted by novel_miR149).

The number of genes in profile 7 was 37, which were the target genes of 11 differentially expressed miRNAs, and the number of genes in profile 5 was 30, which were the target genes of 12 miRNAs. In profile 7, three chloroplast OS genes (i.e., Cluster-86748.59528 was targeted by novel_miR1, and Cluster-86748.60212 and Cluster-86748.60213 were targeted by novel_miR155) and a structural maintenance of chromosome protein (Cluster-97477.0 was targeted by novel_miR1) were detected. In profile 5, Cluster-86748.43155 targeted by novel_miR13 plays a role of protein kinase activity at cell membrane.

### Gene Ontology and Kyoto Encyclopedia of Genes and Genomes Function Analysis of Differentially Expressed Target Genes

**Figure 3:**
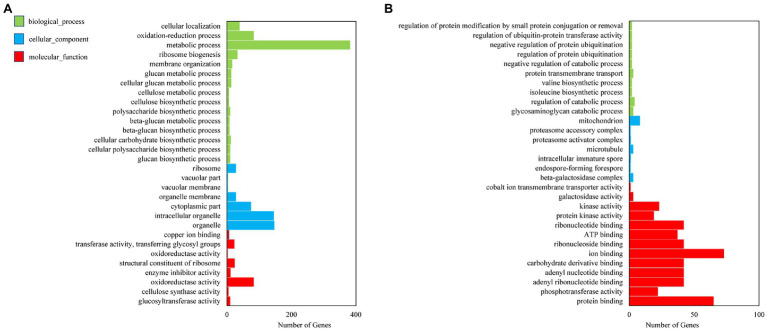
Gene ontology (GO) diagrams of target genes of differentially expressed miRNAs in **(A)** roots and **(B)** leaves. Green, blue, and red colors represent biological processes, cellular components, and molecular functions, respectively.

**Figure 4:**
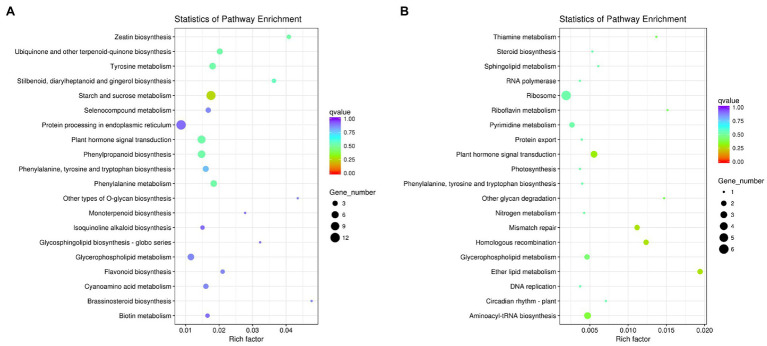
Kyoto Encyclopedia of Genes and Genomes (KEGG) diagrams of target genes of differentially expressed miRNAs in **(A)** roots and **(B)** leaves.

In GO function analysis, two different organs of *M. sinensis* showed different enrichment trends. The function of the target genes of differentially expressed miRNAs in root was annotated with three categories based on their functions, namely, biological processes, cellular components, and molecular functions; among them, biological processes were mainly annotated (Figure 3). For cellular components, these target genes were annotated with cytoplasmic part, intracellular organelle, and organelle. In molecular functions, the gene function is mainly concentrated in oxidoreductase activity, structural constituent of ribosome and transferase activity, and the transferring glycosyl group. In GO function analysis of leaves, molecular functions play a major role in three function analysis, namely, ion binding, ATP binding, and phosphotransferase activity. However, biological processes and cellular components enriched fewer genes. For KEGG analysis, unigenes were searched through the KEGG database to determine the biological processes in which they are involved. Roots were significantly enriched in starch and sucrose metabolism, and leaves were enriched in the ribosome and plant hormone signal transduction (Figure 4).

### qRT-PCR Analysis of Differentially Expressed miRNA

**Figure 5:**
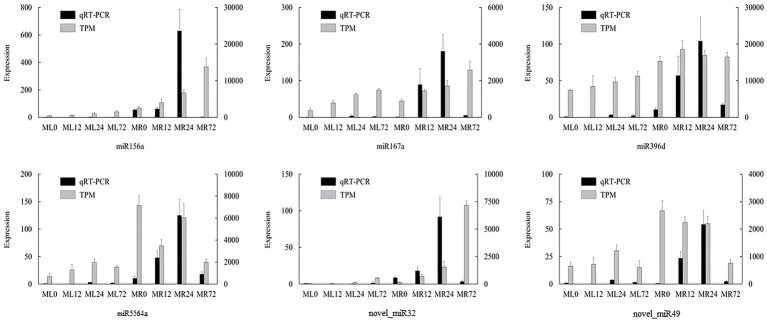
Quantitative real time PCR (qRT-PCR) validation of differentially expressed miRNAs detected by miRNA sequencing. Vertical bars indicate mean values ± SD from three biological replicates.

In order to verify the accuracy of high-throughput sequencing results and validate the expression pattern of miRNAs detected in this study, six differentially expressed miRNAs were randomly selected for qRT-PCR validation. The results showed that the relative expression obtained by qRT-PCR was basically consistent with the TPM values, and most of them have specific expression in a certain tissue (Figure 5). However, there are still some differences between transcriptomic sequencing and qRT-PCR. The expression of miR156a increased continuously in TPM expression, but the qRT-PCR expression of miR156a decreased sharply in MR72, which might be due to the sensitivity and specificity of the two techniques.

## Discussion

Due to the rapid development of industry and the irregular discharge of industrial wastes, heavy metal pollution has become one of the most urgent problems in ecological remediation (He et al., 2005). Previous studies indicated that miRNAs are involved in different heavy metal stress, participating in regulation of gene expression. It has been reported that *Miscanthus* is resistant to heavy metals and can remove heavy metal soil activity (Zhang et al., 2009). However, there are few studies on the extensive identification of Cr-reactive miRNA and its target genes in *M. sinensis*. In this study, 104 conservative miRNAs and 158 nonconservative miRNAs were identified from root and leaf library. Among them, compared with nonconservative miRNAs, most conservative miRNAs had relatively high reads. In addition, the mean number of members of the conservative miRNA family is smaller than that of the nonconservative miRNA family, which is different from previous studies in other species, such as *Fragaria × ananassa* (Li et al., 2013), *M. truncatula* (Zhou et al., 2011), and *Brassica napus* (Chen et al., 2012). In underlying radish plants, 54 conservative miRNAs and 16 novel differentially expressed miRNAs were identified under Cr stress (Liu et al., 2015). In tobacco (*N. tabacum*) roots, 29 conserved miRNAs and 14 novel miRNAs expressed differently under Cr stress (Bukhari et al., 2015). A total of 512 and 568 known miRNAs were identified from Cr treatment and control rice plants, respectively, and 13 conserved miRNAs depicted preferential expression under Cr treatment (Dubey et al., 2020). In total, nine known and 45 novel miRNAs were significantly differentially regulated under Cr stress in this study. Furthermore, there were no known miRNA expression differences in the leaves, and all of them depicted differentially regulated in roots.

Plant response to heavy metal stress was a complex process, including dynamic changes of a large number of miRNAs and target genes. Novel_miR15 and novel_miR22 were two differentially expressed miRNAs in both roots and leaves in this study, which were clustered with miR164a and miR164b, showing an increasing tendency after the treatment. Previous studies indicated that miRNA164 was widely involved not only in plant seed germination, cell division, cell secondary wall formation, meristems formation, flowering, and senescence (Wang et al., 2014), but also in plant response to abiotic stress, such as heavy metal stress (Huang et al., 2009; Zhou et al., 2011). The target genes of miRNA164 family were NAC transcription factors (Xu et al., 2016), and it was found that overexpression of miR164a and miR164b resulted in the reduced expression of *NAC* gene family members, CUC1 and CUC2, in *Arabidopsis*, which were associated with the development of meristematic tissue and the establishment of organ primordium boundaries (Mallory et al., 2004; Nikovics et al., 2006). Accordingly, it was assumed that Cr increased the expression level of these miRNAs, inhibiting the expression of NAC genes related to plant growth and development; thus, the distribution of nutrient and energy could be a turn to plant resistance.

Sulfate transporter (ST) protein had been identified as a major transporter for Cr (VI) uptake. In yeast, the STs (i.e., Sul1 and Sul2) are responsible for Cr (VI) uptake (Chang et al., 2003). Furthermore, a recent study showed that high-affinity STs (i.e., Sultr1 and 2) were involved in Cr (VI) uptake by the roots of *Arabidopsis thaliana* (Xu et al., 2021). An increasing number of studies have demonstrated that miR395 plays an important role in the regulation of STs, and it is crucial for the regulation of sulfate homeostasis (He et al., 2020). In this study, a target gene “Cluster-61280.0” (targeted by miR167a) was identified to regulate the synthesis of Sultr2 transporter. Therefore, miR167a may be a new miRNA involved in the regulation of sulfate proteins under Cr stress. MT protein has metal binding ability and excessive metal ion detoxification (García-Hernández and Taiz, 1998; Jain et al., 2016), and it has high affinity for various heavy metals due to its rich cysteine content (Cobbett and Goldsbrough, 2002). Jain et al. (2016) found that MT gene expression was significantly increased in sugarcane (*Saccharum* spp. *hybrid*) under Cr stress. In the study involving the root of *M. sinensis*, a MT-like protein gene (Cluster-86748.41948) was identified as an important predicted target gene of novel_miR15, which occupied an important position in ion transport function and participated in the detoxification of heavy metal Cr. Besides; novel_miR15 also targeted an ABC transporter protein (Cluster-86748.1466), which was differentially expressed after Cr treatment. The ABC transporter family is one of the largest membrane protein families with a wide variety of species distributed throughout the organism and a variety of transportable substrates, including ions, carbohydrates, lipids, and heavy metals (i.e., As, Cd, As, Hg, and Zn; Long et al., 2011; Wang et al., 2017). Accordingly, it was speculated that novel_miR15 and miR164 had a similar heavy metal detoxification effect based on the clustered results in heat map and predicted target genes of them.

In *M. sinensis*, most differentially expressed miRNAs were identified in roots after Cr treatment, including known miR156 and miR167. For miR156, it was generally identified as a critical factor to help plants to coordinate the relationship between development and stress tolerance and targeted Squamosa promoter binding protein-like genes family. Overexpression of miR156 in *A. thaliana* could increase plant stress tolerance (Cui et al., 2015). In addition, a target of miR156 in *B. napus* encodes glutamyl transferase, an enzyme involved in the formation of chelate states in plant, through which the plant formed chelate states or glutathione tripeptides chelated the heavy metals and eventually sequestered them into vacuoles (Cobbett, 2000; Zhou et al., 2011). Besides, the highly toxic hexavalent Cr ions are redox to the less toxic trivalent Cr ions, which are then transported in a possible detoxification pathway. Cytochrome P450 proteins are important in the oxidative, peroxidative, and reductive metabolisms of numerous endogenous compounds (Joo, 1997). In rice, CYP90D2 encodes C-3 dehydrogenase and has been considered to be involved in brassinosteroid biosynthesis and catabolism (Hong et al., 2003). In this study, cytochrome P450 (Cluster-86748.26078) was also identified as an important target gene of miR156 under Cr stress, which would provide a new insight for further explanation of the detoxification mechanism. MiR167 targeting auxin response factor (*ARF*) gene family also play an important role in regulating root extension and plant shape control (Goetz et al., 2006; Gutierrez et al., 2009). Under salt stress, miR167 was upregulated in both *A. thaliana* (Liu et al., 2008) and *Caragana intermedia* (Zhu et al., 2013), possibly by regulating the expression of *ARFs*, affecting the transcription of auxin response-related genes, thus slowing down the growth and development of plants.

In conclusion, a total of 104 conserved miRNAs and 158 nonconserved miRNAs were identified. Among them, 45 miRNAs were differentially expressed miRNAs in roots and 13 miRNAs were differentially expressed miRNAs in leaves. There were 833 differentially expressed target genes of 45 miRNAs in roots and 280 differentially expressed target genes in leaves. Based on candidate gene annotation and GO and KEGG function analysis, the miR167a, novel_miR15, and novel_miR22 and their targets were potentially involved in Cr transportation and chelation. Besides, miR156a, miR164, miR396d, and novel_miR155 were identified by participating in the physiological and biochemical metabolisms and the detoxification of Cr of plants. These results provided a valuable reference original insight into the role of miRNAs on the metabolic fingerprinting of Cr, regarding the targeted miRNAs. In future, the identified miRNAs and target genes will provide a fundamental process toward understanding miRNA-mediated regulatory networks of *M. sinensis* response under Cr (VI) stress.

## Data Availability Statement

The original contributions presented in the study are publicly available. This data can be found here: miRNA-seq data were deposited into NCBI database under accession number PRJNA595773, and the transcriptome sequence data were deposited into NCBI database under accession number PRJNA594536.

## Author Contributions

XZ and GN contributed to the experiment design and funding acquisition. MZ, ZL, JZ, and AL performed the material preparation and data collection. GN, MZ, XW, and JC analyzed the data. GN, ZL, MZ, and AL wrote the first draft of the manuscript. All authors commented on previous versions of the manuscript, and read and approved the final manuscript.

### Conflict of Interest

The authors declare that the research was conducted in the absence of any commercial or financial relationships that could be construed as a potential conflict of interest.

## Footnotes

^1^http://www.mirbase.org/ftp.shtml

^2^http://rfam.sanger.ac.uk/search/

^3^https://magic.novogene.com

^4^http://www.omicshare.com/tools
